# Impact of inherent variability and experimental parameters on the reliability of small animal PET data

**DOI:** 10.1186/2191-219X-2-26

**Published:** 2012-06-09

**Authors:** Marianne Isabelle Martic-Kehl, Simon Mensah Ametamey, Malte Frederick Alf, Pius August Schubiger, Michael Honer

**Affiliations:** 1 Center for Radiopharmaceutical Sciences of ETH, Institute of Pharmaceutical Science of ETH Zürich, Wolfgang-Pauli-Str. 10, Zurich, 8093, Switzerland; 2 Collegium Helveticum, ETH and USZ, Schmelzbergstr. 25, Zürich, 8092, Switzerland; 3 CNS Molecular Imaging, F. Hoffmann-La Roche Ltd., Grenzacherstrasse 124, Basel, 4070, Switzerland

**Keywords:** Inter-animal variability, Intra-animal variability, Homogenization, Reproducibility, External validity

## Abstract

**Background:**

Noninvasive preclinical imaging methodologies such as small animal positron emission tomography (PET) allow the repeated measurement of the same subject which is generally assumed to reduce the variability of the experimental outcome parameter and to produce more robust results. In this study, the variability of tracer uptake in the rodent brain was assessed within and between subjects using the established radiopharmaceuticals ^18^F-FDG and ^18^F-fallypride. Moreover, experimental factors with potential impact on study outcome were elicited, and the effect of their strict homogenization was assessed.

**Methods:**

Brain standardized uptake values of rodents were compared between three PET scans of the same animal and scans of different individuals. ^18^F-FDG *ex vivo* tissue sampling was performed under variation of the following experimental parameters: gender, age, cage occupancy, anesthetic protocol, environmental temperature during uptake phase, and tracer formulation**.**

**Results:**

No significant difference of variability in ^18^F-FDG or ^18^F-fallypride brain or striatal uptake was identified between scans of the same and scans of different animals (COV = 14 ± 7% vs. 21 ± 10% for ^18^F-FDG). ^18^F-FDG brain uptake was robust regarding a variety of experimental parameters; only anesthetic protocols showed a significant impact. In contrast to a heterogenization approach, homogenization of groups produced more false positive effects in ^18^F-FDG organ distribution showing a false positive rate of 9% vs. 6%.

**Conclusions:**

Repeated measurements of the same animal may not reduce data variability compared with measurements on different animals. Controlled heterogenization of test groups with regard to experimental parameters is advisable as it decreases the generation of false positive results and thus increases external validity of study outcome.

## Background

Small animal positron emission tomography (PET) is a frequently used methodology to investigate rodent models of healthy and diseased states. Preclinical PET has been revolutionized with the development of dedicated small animal PET scanners [1-3]. Noninvasive imaging methods such as PET are considered to give more reliable results in longitudinal follow-up studies where animals can be used as their own control compared with studies where test and control animals are not identical [1,4]. Additionally, strict homogenization of experimental parameters reduces variability within test groups and is therefore believed to increase reliability of animal experiments [5,6]. Nevertheless, recent investigations on mouse behavior revealed that the rate of false positive outcome was significantly higher under homogenized conditions compared with an approach investigating pseudo-heterogenized groups [7]. Homogenization proved to decrease the reproducibility of results among test groups. It is therefore suggested to heterogenize test groups in a controlled manner in order to increase external validity of results, i.e., the applicability of a result to other conditions, populations, or species [7-10]. On the other hand, only homogenization of test groups guarantees the detection of environmental influences on experimental outcome [6].

In this study, two representatives of different PET tracer categories were investigated as exemplary tracers. The glucose analogue ^18^F-FDG is the most frequently used PET tracer in the clinic and a ‘metabolic’ tracer. Previous studies in the field of small animal PET have shown that the following experimental parameters crucially impact biodistribution of ^18^F-FDG: anesthetic agents, carrier gas, fasting, ambient temperature, and injection type [11-16]. The dopamine D_2_ receptor ligand (S)-N-[(1-allyl-2-pyrrolidinyl)methyl]-5-(3-^18^F-fluoropropyl)-2,3-dimethoxybenzamide, ^18^F-fallypride, is a representative of a radioligand binding reversibly to a site on a neurotransmitter receptor. ^18^F-fallypride shows excellent binding properties (high affinity and selectivity) and imaging characteristics for the visualization of the dopamine D_2_ receptor subtype [17-19].

The main focus of the inevitable variability determination was on the brain for ^18^F-FDG and on the striatum in the case of ^18^F-fallypride. To investigate variability of tracer uptake, it was important to choose an organ, which can guarantee a certain intrinsic stability. A detailed study on ^18^F-FDG tumor uptake variability showed 15.4 ± 12.6% variability between two scans performed with a 6-h delay on the same day [20]. Even though ^18^F-FDG is mainly used for tumor studies, our study focuses on the brain, due to less expected intrinsic variability.

In the first part of this study, the inherent variability of ^18^F-FDG brain and ^18^F-fallypride striatum uptake within and between individual animals was assessed in a test-retest setup using a highly standardized protocol. The second part was focusing on the determination of experimental factors, which might essentially impact the outcome of ^18^F-FDG and ^18^F-fallypride rodent studies. Finally, in a third part, homogenization and heterogenization of protocols were compared in terms of gender differences of ^18^F-FDG biodistribution for an experimental setting with varying age of test animals and cage occupancy. It was observed that animal age often varies not within one experimental group, but often between different individual experiments. Purposely introducing variation to an experimental setup might well feasibly be achieved with animals of different age. The same holds for the parameter cage occupancy. The latter often varies between as well as within experimental setups.

The aim of this study was the assessment of suitable small animal PET imaging protocols leading to minimal inevitable tracer uptake variability and therefore of an estimation of minimally detectable effect sizes in preclinical PET studies. Furthermore, it was an attempt to transpose the findings in behavioral experiments regarding the heterogenization in experimental setup to a more presumably robust field of animal experimentation, i.e., PET tracer tissue distribution. The translation of preclinical results to the clinical setting is often problematic, and it can be assumed that this is the case in PET research as well.

## Methods

### Animal preparation

Healthy animals were purchased from Charles River, Sulzfeld, Germany. The investigated strains were Naval Marine Research Institute (NMRI) mice, C57Bl/6J mice, and Sprague Dawley (Crl:CD(SD)) rats with gender, age, and group size as listed in Table 1.

**Table 1:** Test parameters of individual experiments

			^**18**^**F tracer**		**Anesthetic protocol (min)**			**Ambient temperature (°C)**		**10% EtOH**		**Gender**		**Age (weeks)**			**Cage density (*n*)**		
**Experiment**	**Strain**	***n***	**FDG**	**fallypride**	**Control (6)**	**40**	**60**	**RT**	**33**	**Yes**	**No**	**M**	**F**	**5**	**7 to 10**	**14**	**2**	**3 to 8**	**13**
Test-retest study 1	SD rat	6	x			x		x			x	x			x			x	
Test-retest study 2	SD rat	6	x			x		x			x	x			x			x	
Test-retest	NMRI	6		x		x		x			x	x			x			x	
	C57BL/6 J	7		x		x		x			x	x			x			x	
Anesthetic impact	SD rat	6	x		x			x				x			x			x	
	SD rat	7	x			x		x				x			x			x	
	SD rat	7	x				x	x				x			x			x	
Temperature/EtOH impact	NMRI	8	x			x		x			x	x			x			x	
	NMRI	8	x			x			x		x	x			x			x	
	NMRI	8	x			x		x		x		x			x			x	
	NMRI	8	x			x			x	x		x			x			x	
Homogenization/heterogenization	NMRI	10	x			x		x			x	x		x			x		
	NMRI	10	x			x		x			x	x				x	x		
	NMRI	11	x			x		x			x	x		x					x
	NMRI	11	x			x		x			x	x				x			x
	NMRI	9	x			x		x			x		x	x			x		
	NMRI	8	x			x		x			x		x			x	x		
	NMRI	11	x			x		x			x		x	x					x
	NMRI	12	x			x		x			x		x			x			x

x, Experimental parameters applied for each individual animal group; EtOH, ethanol; C57Bl/6J mice; NMRI, Naval Marine Research Institute mice; SD rat, Sprague-Dawley rat.

Animal care and experimental procedures were performed in accordance with and approved by the Swiss Federal Veterinary Office. Animals were kept in standard cages (groups of 2 to 13 animals per cage) in a Scantainer (Scanbur, Denmark) equipped with a filter cover. Three types of cages were used for animal housing. Pairs of mice were kept in type II cages (16 × 22 × 14 cm^3^ (width × length × height); Tecniplast, Hohenspeissenberg, Germany). Medium groups of three to eight mice were housed in type III cages (22 × 37 × 15 cm^3^). Groups of up to 13 mice as well as all groups of rats were kept in type IV cages (33 × 55 × 25 cm^3^). Ambient temperature was set to 23°C, and air humidity was between 50% and 85%. Free access to food (*Alleinfuttermittel* for rats and mice, KLIBA NAFAG, Kaiseraugst, Switzerland)**,** and water was allowed throughout all experiments. A light to dark cycle of 12 h (dark phase, 6 p.m. to 6 a.m.) was maintained throughout all studies. Animal monitoring and cage changes were performed weekly by the experimenter or a professional animal caretaker. Experiments were started between 7 and 9 a.m. involving always the same two experimenters.

### Radiotracer application

^18^F-FDG was obtained from the commercial ^18^F-FDG production of the University Hospital Zurich in batches of 1 to 2 GBq. The radiosynthesis of ^18^F-fallypride was performed according to the protocol of Mukherjee et al. [14]. The radioligand was produced in batches of 500 to 5,000 MBq, with activity concentrations of 500 to 2,000 MBq/mL and specific activities between 54 and 260 GBq/μmol at the end of synthesis. Radiotracer injection into rats was performed using the Vasofix®Braunüle® (Braun AG, Melsungen, Germany) catheter. Tail vein injections of approximately 15 MBq ^18^F-FDG in 300 μL of physiological NaCl solution (Braun AG) were followed by rinsing the catheter with 150 μL of physiological NaCl solution. In mice, approximately 15 MBq ^18^F-FDG in 100 μL of physiological NaCl solution were directly injected into the tail vein. Likewise, ^18^F-fallypride was injected into mice via direct tail vein injection of 100 μL. In order to keep the injected cold mass constant over each test day, decreasing radioactive doses of ^18^F-fallypride were injected over each test day (between 18 and 2 MBq). Over all test days, the injected cold mass of ^18^F-fallypride was between 20 to 130 ng corresponding to 1.5 to 9.0 nmol/kg body weight.

### Small animal PET imaging

All PET experiments were performed at the Animal Imaging Center (Center for Radiopharmaceutical Sciences, ETH Zurich) using the dedicated 36-module eXplore Vista-PET/CT tomograph (GE Healthcare, Waukesha, WI, USA) with a maximum resolution of higher than 2 mm full width at half maximum and a field of view (FOV) with an axial length of 48 mm and a diameter of 67 mm [21]. Animals underwent one bed position scans with the brain in the center of the FOV.

#### PET protocol 1: anesthesia induction after tracer injection

Animals were restrained and injected 30 min (^18^F-FDG) and 20 min (^18^F-fallypride) before scan start. The urinary bladder was emptied by pressing the lower abdomen before induction of anesthesia 10 min before scan start, and animals were then fixed on the bed of the scanner. Isoflurane was used as anesthetic agent of choice with oxygen/air (50%/50%) as carrier gas. Data were acquired from 30 to 60 min post injection (p.i.) for ^18^F-FDG emission scans and from 20 to 60 min p.i. for ^18^F-fallypride emission scans (energy window 250 to 700 keV). Images were reconstructed using a two-dimensional-ordered subset expectation maximization algorithm (2 iterations, 16 subsets) with scatter and random correction; no attenuation correction was performed. Voxel size of reconstructed images was 0.3875 × 0.3875 × 0.775 mm^3^.

#### PET protocol 2: anesthesia induction before tracer injection

Animals were anesthetized, and an injection catheter (Vasofix®Braunüle® (Braun AG), 22 G, 0.9 × 25 mm) was placed in a lateral tail vein and rinsed with 100 μL Heparin solution (Heparin-Na (Bichsel AG, Interlaken, Switzerland), 25,000 I.E./5 mL) before animals were positioned on the scanner bed. Tracer injection and PET scan start were performed simultaneously. Data was acquired from 0 to 60 min p.i. for ^18^F-FDG emission scans in the list mode format (energy window 250 to 700 keV). Data were split into two time frames of 30 min each, and images were reconstructed as described above. For dynamic scanning protocol, only the second 30 min frame was further investigated.

Reconstructed images were inspected in coronal, sagittal, and transverse planes throughout the reconstructed volume. Region of interest (ROI) analysis was performed with the biomedical image quantification software PMOD (Pmod Technologies Ltd, Adliswil, Switzerland) [22]. ROIs were drawn manually using the whole brain for ^18^F-FDG studies and striatum for ^18^F-fallypride studies. Tracer uptake was quantified as standardized uptake value (SUV) by normalizing the average activity concentration (counts per second per milliliter) of each volume of interest (VOI) to the injected dose per body weight (MBq/kg).

### Measurement of physiological parameters

Body temperature of all animals under isoflurane anesthesia was monitored using a rectal temperature sensor. A stream of warm air was blown through the scanner tube to avoid hypothermia during anesthesia and to keep body temperature constantly between 35°C and 37°C. Depth of anesthesia monitoring was achieved by sensing the breath rate with an abdominal breathing belt (rats) or by visual breath rate counting (mice). The breath rate was kept constant at a rate of approximately 60 breaths/min by adjustment of anesthesia.

### Assessment of test-retest variability of ^18^F-FDG whole brain and ^18^F-fallypride striatum uptake

Three static PET scanning experiments were performed for each individual animal of a group to assess the variability between brain scans of the same animal (intra-animal variability) and scans of different individuals (inter-animal variability). Between scans, animals underwent one week of recovery. ^18^F-FDG scans were performed with SD rats (male, 200 to 300 g, *n* = 6) and ^18^F-fallypride scans with NMRI mice (male, 34 to 39 g, *n* = 7) and C57Bl/6 J mice (male, 18 to 26 g, *n* = 7). The ^18^F-FDG study was performed twice using two independent batches of animals, referred to as study 1 and study 2. Variability of tracer uptake to the brain (^18^F-FDG) or striatum (^18^F-fallypride) was calculated as coefficient of variation (COV), corresponding to the relative standard deviation of the mean.

### Influence of experimental parameters on ^18^F-FDG and ^18^F-fallypride biodistribution

To determine the impact of different experimental parameters on ^18^F-FDG biodistribution, animals underwent PET scanning according to protocols 1 or 2. Subsequent to PET imaging, animals were euthanized by decapitation at approximately 61 min p.i. Organs and tissues were collected; the wet weights were determined, and the samples were measured by classical gamma counting (Wallac 1480 Wizard, PerkinElmer Instruments, Boston, MA, USA). Radiotracer uptake was expressed as percentage of injected dose per gram tissue normalized to the body weight of the animals (normalized percent injected dose per gram (norm. %ID/g)) and calculated as follows: sample activity (counts per minute)·100/[injected dose (counts per minute)·sample weight (grams)]/body weight (kilograms). Individual test parameters of experiments are listed in Table 1.

#### Anesthetic protocols (male SD rats, 220 to 280 g, n = 6)

The impact of anesthetic protocols on ^18^F-FDG organ and tissue uptake was investigated by applications of isoflurane anesthesia of different durations (0 to 61 min p.i. for dynamic PET scanning (PET protocol 2), 20 to 61 min p.i. for static PET scanning (PET protocol 2), and 55 to 61 min p.i. as control conditions).

#### Temperature (male NMRI mice, 18 to 46 g, n = 8)

Animals underwent a static PET scanning protocol (PET protocol 2). Temperature control during ^18^F-FDG uptake phase (0 to 20 min p.i.) was achieved by placing the animal under an infrared lamp subsequent to tracer injection. The temperature at the position of the animal was 33°C. Control animals were kept in the same room at 23°C.

#### EtOH in the administered tracer solution (^18^F-FDG: male NMRI mice, 18 to 46 g, n = 8; ^18^F-fallypride: male C57Bl/6 J mice, 20 to 25 g, n = 8)

^18^F-FDG or ^18^F-fallypride were injected in physiological saline solution containing 10% of EtOH (*v*/*v*). This amount corresponds to a blood concentration of approximately 4‰ ethanol for a mouse of average size. Tracer solutions injected to control animals did not contain 10% EtOH. Experimental handling, data collection, and interpretation were performed in a blinded fashion.

### Analysis of homogenization vs. pseudo-heterogenization

**Figure 1:**
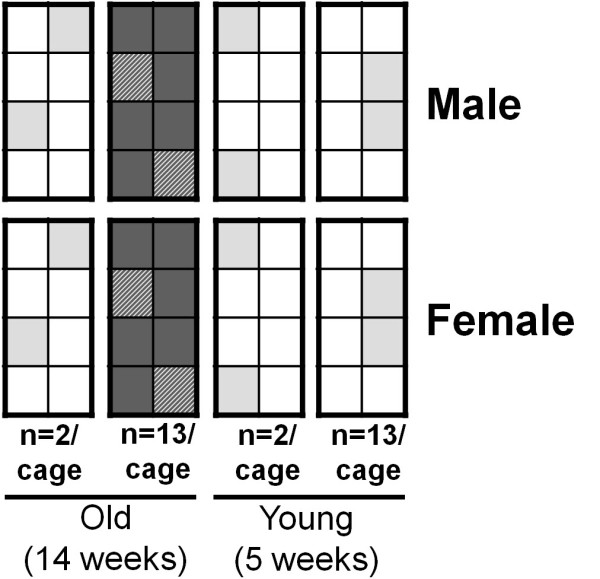
**Experimental design of homogenization vs. heterogenization.** The scheme of the experiment is in analogy to the study of Richter et al. [7]; 8 × 8 animals underwent ^18^F-FDG scans according to PET protocol 1. Varying experimental parameters were gender, age, and cage occupancy of the animals (*n* = 2 or *n* = 13 per cage). Animal groups were divided according to gender. This setup allowed for four strictly homogenized test group comparisons with the only parameter varying being gender (one example of such a comparison pair is indicated in dark gray). On the other hand, by reordering the same data into 8 × 8 randomized groups, four comparisons of such pseudo-heterogenized groups were performed (indicated by the light grey squares).

Comparison of homogenization with heterogenization of test groups was performed according to the study of Richter and co-workers (Figure 1) [7]. ^18^F-FDG scans according to PET protocol 1 were performed with eight groups of NMRI mice under different homogenized conditions. Animals were sacrificed subsequent to the PET scan (61 min p.i.); 19 organs and tissue samples were collected and measured by classical gamma counting. The data of all 4 × 8 animals of each gender were pooled, and the ‘true’ gender differences in ^18^F-FDG uptake were determined. Gender differences in ^18^F-FDG tissue distribution were then assessed by comparing four pairs of highly homogenized test groups (an example of one homogenized pair is indicated in dark grey, Figure 1). Each pair was statistically analyzed using a two-tailed Student's *t* test with subsequent Bonferroni correction. The identified gender differences were compared with the ones determined in the pooled setup, and the number of false positive differences was assessed. This step was repeated for four pairs of ‘pseudo-heterogenized’ groups by randomly distributing the same data to eight ‘new’ groups (indicated in light grey, Figure 1). The variability (COV) of ^18^F-FDG tissue uptake between and within groups (2 × 4 homogenized groups and 2 × 4 pseudo-heterogenized groups) was calculated and compared using a nonparametric Wilcoxon signed rank test. By adapting the significance level *α* for the heterogenized data, the false negative rate was brought to the same level as the one arising from the homogenized setup, thereby achieving direct comparability of false positive rates via a *t* test.

### Statistical analysis

Figures show mean ± SD, unless stated otherwise. Differences among experimental groups in SUVs and percent injected dose per gram values of the various tissues investigated were statistically evaluated by analysis of variance (ANOVA) and subsequent *post hoc* Tukey tests for comparisons of three or more groups and by two-tailed Student's *t* tests of unpaired samples for comparisons of two groups. Intraindividual variability COVs were statistically compared using a paired sample Student's *t* test. Statistical significance was set at the 95% level, and Bonferroni correction was applied where required. All statistical analyses were performed using the computer software SPSS 15.0 version for Windows (SPSS Inc. Chicago, Illinois).

## Results and discussion

### Results

#### Test-retest variability of ^18^F-FDG brain uptake and striatal uptake of ^18^F-fallypride

**Figure 2:**
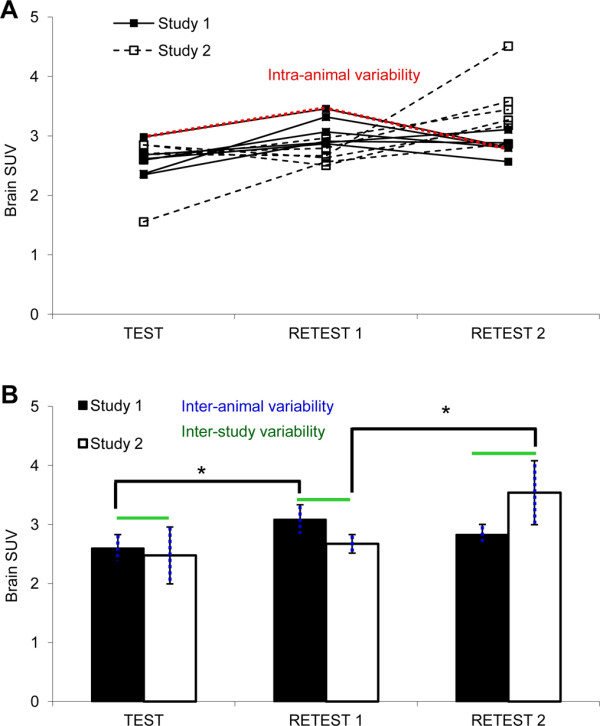
**Different variability types.** (**A**) Different types of variability of ^18^F-FDG brain SUV in male Crl:CD(SD) rats (*n* **=** 6). Each data point corresponds to the ^18^F-FDG brain SUV of one individual animal. Intra-animal variability is the variability between three brain scans of one individual (indicated in red). (**B**) Column bar representation of the data from (A). Inter-animal variability is the variability between brain SUVs of different individuals acquired on the same test day (represented by the SD, indicated in blue). Inter-study variability is the variability of ^18^F-FDG brain SUVs between two independent test groups acquired under exactly the same experimental conditions (indicated in green); *P < 0.05.

The different variability types investigated are illustrated in Figure 2A. Inter- and intra-animal variability of ^18^F-FDG brain uptake did not differ significantly: 8 ± 2% (inter-animal COV) compared to 10 ± 4% (intra-animal COV) for the first study, and 14 ± 7% (inter-animal COV) compared to 21 ± 10% (intra-animal COV) for the second study. By trend, intra-animal variability was greater than inter-animal variability. Repetition of the test-retest setup resulted in an inter-study variability of 10 ± 6% for ^18^F-FDG brain uptake. ^18^F-FDG brain uptake of retest 1 compared to either test or retest 2 proved to be significantly different (16% and 24%, *P* < 0.05; Figure 2B).

**Figure 3:**
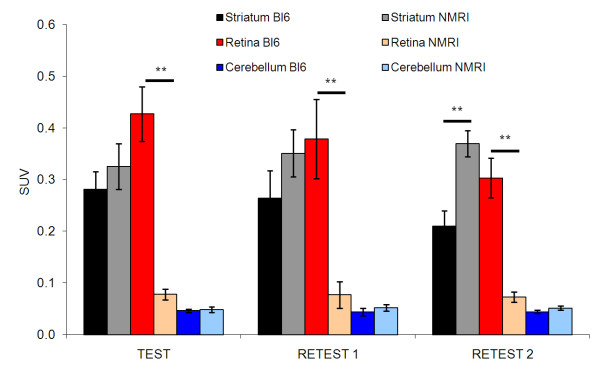
^**18**^**F-fallypride SUV in striatum, retina, and cerebellum.** Black, striatum; red, retina; blue, cerebellum. NMRI mice (light colors): *n* = 6, C57Bl/6 J mice (dark colors): *n* = 7; ***P < 0.001.

Inter- and intra-animal variability of ^18^F-fallypride striatum uptake did not differ significantly: 16 ± 4% (inter-animal COV) compared to 23 ± 8% (intra-animal COV) for C57Bl/6 J mice, and 11 ± 4% (inter-animal COV) compared to 9 ± 8% (intra-animal COV) for NMRI mice. Likewise, ^18^F-fallypride striatum uptake between individual test days did not differ significantly, whereas NMRI mice tended to accumulate more ^18^F-fallypride in the striatum than C57Bl/6 J mice; on the third test day, the difference was significant (Figure 3). Strain differences also occurred concerning accumulation of ^18^F-fallypride in the eye region. The very high ^18^F-fallypride accumulation in the eye region observed in C57Bl/6 J mice was mainly due to a non-blockable uptake or binding to the retina (autoradiography experiments, unpublished results). This phenomenon was not observed in NMRI mice; ^18^F-fallypride accumulation in the eye region was in the background range, comparable with cerebellum uptake (Figure 3).

#### Impact of experimental parameters on ^18^F-FDG and ^18^F-fallypride ex vivo tissue distribution

The anesthetic protocol (PET protocol 1 vs. 2) had a significant impact on ^18^F-FDG uptake in several tissues. ^18^F-FDG brain uptake of animals anesthetized during the whole tracer uptake phase (protocol 2) was reduced by 27% compared to animals investigated under protocol 1 conditions (*P* < 0.01). Protocol 1 resulted in a 17% reduction of brain uptake compared with the control group which was not scanned (short anesthesia of 6 min for humane decapitation; *P* < 0.01). ^18^F-FDG muscle uptake showed a similar pattern as brain uptake with highest uptake under control conditions (*P* < 0.01). Contrary to these findings, ^18^F-FDG blood concentration was lowest in the control and highest in the test group investigated according to protocol 2 (*P* < 0.01), as shown in part A of Table 2. An ambient temperature of 33°C during the first 20 min after ^18^F-FDG injection resulted in 40% decreased ^18^F-FDG brown fat tissue uptake compared with control animals at room temperature (r.t.) or animals with 10% ethanol co-administration at r.t. (not significant). Ethanol seemed to counteract the reduced ^18^F-FDG brown fat tissue uptake observed at 33°C ambient temperature alone. Furthermore, animals receiving 10% ethanol showed a 34% decrease in muscle uptake compared with the test group kept at higher ambient temperature (*P* < 0.05, part B of Table 2). *Ex vivo* biodistribution of ^18^F-fallypride was not significantly influenced by 10% ethanol in the administered tracer solution. Nevertheless, there was a trend towards increased ^18^F-fallypride accumulation in the striatum under ethanol conditions (16%, not significant, part C of Table 2). Gender and age of test animals proved to significantly impact ^18^F-FDG uptake to certain peripheral organs, but not to the brain. More than double the amount of ^18^F-FDG was accumulated in the fat of females compared with males (*P* < 0.01), whereas ^18^F-FDG blood concentration was 33% higher in males (*P* < 0.01, part D of Table 2). Older animals accumulated 13% less ^18^F-FDG in their bone marrow (*P* < 0.05) and 28% more ^18^F-FDG in their Harderian glands (*P* < 0.01) compared with the younger mice. The potential influence of combined effects of two or three of the investigated parameters was excluded by performance of a three-way ANOVA where no significant differences due to multiple parameters were confirmed.

**Table 2:** **Influence of experimental parameters on tissue distribution of**^**18**^**F-FDG or**^**18**^**F-fallypride expressed as normal %ID/g (mean ± SD)**

**Part**	**Experimental parameter**	**Organ**			
A	Anesthetic protocol (^18^F-FDG)	Brain	Muscle	Blood	
	Control	0.31 ± 0.04	0.066 ± 0.026	0.015 ± 0.006	
	Dynamic	0.19 ± 0.04**	0.028 ± 0.006**	0.051 ± 0.009**	
	Static	0.26 ± 0.02**	0.04 ± 0.006**	0.033 ± 0.004**	
B	Ambient temperature/10% EtOH (^18^F-FDG)	Brown fat tissue	Muscle		
	r.t. (23°C)	0.30 ± 0.18	0.097 ± 0.038		
	33°C	0.18 ± 0.07 (n.s.)	**0.119 ±** 0.016*		
	r.t./10% EtOH	0.37 ± 0.14	**0.08 ±** 0.023*		
	33°C/10% EtOH	0.40 ± 0.19	0.089 ± 0.018		
C	10% EtOH (^18^F-fallypride)	Striatum			
	r.t. (23°C)	0.25 ± 0.03 (n.s.)			
	33°C	-			
	r.t./10% EtOH	0.28 ± 0.03 (n.s.)			
	33°C/10% EtOH	-			
D	Gender (^18^F-FDG)	Liver	Blood	Fat	Reproductive organs
	Female	0.018 ± 0.005	0.01 ± 0.003	0.024 ± 0.015	0.052 ± 0.017
	Male	0.022 ± 0.006*	0.015 ± 0.005**	0.011 ± 0.009**	0.041 ± 0.009**
E	Age (^18^F-FDG)	Bone	Harderian gland	Urine	
	5 weeks	0.097 ± 0.016	0.45 ± 0.12	3.4 ± 2.3	
	14 weeks	0.084 ± 0.022*	0.63 ± 0.22**	1.9 ± 1.1*	

n.s., Not significant. Part A is the influence of anesthesia duration on ^18^F-FDG distribution. **P < 0.01 for comparison with control condition. *Ex vivo* biodistribution of ^18^F-FDG in Crl:CD(SD) rats (male), *n* = 6. Control: anesthesia from 55 to 61 min p.i.; dynamic: anesthesia from 0 to 61 min p.i.; static: anesthesia from 20 to 61 min p.i. Part B is the influence of ambient temperature and ethanol application on ^18^F-FDG distribution. *P < 0.05 for comparison between conditions in bold. *Ex vivo* biodistribution of ^18^F-FDG in NMRI mice (male) under control conditions, ambient temperature control between 0 to 20 min p.i., 10% ethanol in the administered tracer solution (10 μL ethanol) and a combination of the two; *n* = 8. Part C is the influence of ethanol in the tracer solution on ^18^F-fallypride distribution. *Ex vivo* biodistribution of ^18^F-fallypride in C57Bl/6 J mice (male), *n* = 8. Comparison of mice receiving 10% ethanol in the administered tracer solution (corresponding to 10 μL ethanol) with control animals. Part D is the influence of gender, age, and cage occupancy on ^18^F-FDG biodistribution. *P < 0.05, **P < 0.01. *ex vivo* biodistribution of ^18^F-FDG in male and female NMRI mice (*n* = 38 for males, *n* = 44 for females). Part E is the *ex vivo* biodistribution of ^18^F-FDG in 14 weeks and 5 weeks old NMRI mice (*n* = 40 for ‘old’ animals and *n* = 42 for ‘young’ animals). *P < 0.05, **P < 0.01 for comparison between male and female or 5 and 14 weeks old animals, respectively.

#### Homogenization vs. pseudo-heterogenization

In analogy to the study of Richter and co-workers [7], it was investigated whether strict homogenization of experimental test groups in small animal ^18^F-FDG PET might lead to reduced reproducibility of results compared with heterogenized test groups. Each group was strictly homogenized to the parameters: gender, age, and cage occupancy (Figure 1). Therefore, ^18^F-FDG tissue uptake was determined in eight test groups. In the first step, animals with the same gender were pooled into one group of 4 × 8 animals in order to determine true gender differences in ^18^F-FDG tissue uptake. Such true differences were found for 2 tissues (blood and fat) out of 19 (2 positive findings vs. 17 negative ones). Having assessed the true positives, gender differences of ^18^F-FDG uptake in 19 different organs and tissue samples were determined by comparing pairs of groups only differing in gender. Inter- and intra‐group variability (COV) of ^18^F-FDG tissue uptake was determined and compared with the results from the pseudo-heterogenized setup (Figure 1). The intra-group ^18^F-FDG tissue uptake COVs were 17% greater for the heterogenized groups compared with those of the homogenized groups (*P* < 0.01). On the other hand, the variability between different test groups was 13% smaller for the heterogenized setup (*P* < 0.05) as shown in Table 3.

**Table 3:** **Influence of homogenization and heterogenization on**^**18**^**F-FDG organ uptake variability**

**Variability (COV)**	**Homogenized parameters**	**Heterogenized parameters**
Inter‐group	32 ± 2%	28 ± 2%*
Intra‐group	31 ± 1%	35 ± 1%**

*P < 0.05, **P < 0.01. Inter- and intra-group variability of ^18^F-FDG uptake for all organs measured (mean of COVs ± SEM of homogenized groups vs. heterogenized groups, in percent).

The false positive rate of homogenized and pseudo-heterogenized results was determined by comparison with true gender differences of ^18^F-FDG tissue uptake that resulted to pooled data of the same gender. It was investigated whether strict homogenization leads to increased false positive rates of results. Homogenized test groups produced a false positive rate of 9 ± 5% (mean ± SEM), whereas heterogenized test groups resulted in 2 ± 3% false positives (not significant, Table 4). The two false positive rates determined at a significance level *α* of 5% cannot be compared with each other directly due to power differences. The power and the significance level *α* of a study are dependent on each other (i.e., if alpha increases, the power increases as well). In addition to that, study power increases with increasing homogenization as homogenization reduces variability of data. Therefore, the false negative rate of the two setups needed to be compared as well (study power = 1 − false negative rate). The false negative rate of the homogenized setup was significantly lower than one of the heterogenized study (63% vs. 100%, *P* < 0.05; Table 4). The significance level *α* of the pseudo-heterogenized setup was therefore adapted from 0.05 to 0.38 in order to achieve a comparable study power for both setups (power 37%). The recalculated (using the adapted *α*) false positive rate of heterogenized samples was 6 ± 6% compared to 9 ± 5% for the homogenized ones (not significant, Table 4).

**Table 4:** Comparison of false positive and false negative rates between homogenized setup and pseudo-heterogenized setup

	**Homogenized test groups**	**Heterogenized test groups (*α*** **= 0.05)**	**Heterogenized test groups (*α*** **= 0.05)**
False positive rate (%)	9 ± 5	2 ± 3	6 ± 6
False negative rate (%)	63	100*	63

*P < 0.05. For heterogenized groups, *α* was adapted from 0.05 to 0.38 in order to achieve the same study power as in the homogenized setup; *n* = 4; mean of false positive/negative rates ± SEM in percent.

### Discussion

For ^18^F-FDG experiments, the brain was chosen as the main organ of interest as it was assumed that it is a well identifiable organ in small animal PET scans with reasonably low inherent variability of metabolism between individual animals. It is important to bear in mind that the resulting data is not valid for other tissues or organs of the mouse or rat body to the same extent. Nevertheless, it might be reasonable to assume that the inherent tracer uptake variability to different organs or tissue would either be in a similar range or even higher. The reproducibility of ^18^F-FDG rat brain PET scans was not found to be improved by repeated measurement of the same animal. The variability of ^18^F-FDG brain uptake and of striatal ^18^F-fallypride accumulation between scans of the same animal (intra-animal variability) compared with scans of different individuals (inter-animal variability) was higher by trend (not significant). Furthermore, measuring the same group of animals repeatedly produced significantly different brain SUVs on different test days. This might be due to the fact that these animals have already experienced radiotracer injection, anesthesia, and general experimental handling, which might impact the metabolism of individual animals, e.g., the variability in body temperature during the PET scan. Body temperature of animals under anesthesia was maintained between 35°C and 37°C. This temperature variation was in a similar range as reported by Fueger et al. [11]. In addition, animals were one week older at the time point of the retest. The most plausible explanation, however, would be that the determined significances are false positives as it is known that strict standardization leads to a clear increase of the false positive rate [7]. It is therefore advisable to use heterogenized experimental conditions in order to avoid high false positive rates. Controlling heterogenized conditions is very important as certain parameters might crucially impact study outcome, and such parameters should be standardized to reach a satisfactory study power.

Several parameters with a potential impact on ^18^F-FDG biodistribution were investigated in detail. Of all tested parameters, anesthetic protocols proved to influence ^18^F-FDG brain uptake the most. Therefore, it is suggested to standardize anesthetic protocols to produce reliable small animal ^18^F-FDG brain PET data. Results revealed that duration of wakefulness during the first phase of tracer uptake was directly related to ^18^F-FDG brain uptake, which is presumably due to a decreased cerebral metabolic glucose rate under isoflurane anesthesia [23]. These results confirm similar findings of the impact of isoflurane on ^18^F-FDG brain uptake [11,12,14]. ^18^F-FDG muscle uptake showed a similar decrease with anesthesia duration. This effect is most probably due to the lack of motion during anesthesia. On the other hand, blood content of ^18^F-FDG was clearly higher, the longer the duration of anesthesia; this effect might arise from reduced elimination of ^18^F-FDG.

Experimental parameters such as ambient temperature, gender, and age significantly altered ^18^F-FDG uptake to liver, fat tissue, Harderian glands, and bone marrow. The reduced brown fat tissue uptake of ^18^F-FDG at an increased ambient temperature is in line with the findings of Fueger and co-workers [11]. In this study, mice were kept at ambient temperatures of thermoneutrality, which is between 30°C and 34°C for mice. In the thermoneutrality zone, brown fat dependent thermoregulation does not take place. While it is obvious that increased ambient temperature during ^18^F-FDG uptake leads to a decreased brown fat uptake compared with animals kept at room temperature, the impact of age on bone marrow and Harderian gland uptake is less apparent. It seems that young animals might have an increased glucose turnover rate in bone marrow. ^18^F-FDG fat tissue uptake was higher in females compared with that in males, which might be explained by a higher generation of fat deposits in females. An ethanol concentration of 10% in the tracer solution did generally not impact ^18^F-FDG or ^18^F-fallypride tissue distribution significantly, but the reduced ^18^F-FDG brown fat uptake at 33°C ambient temperature was counteracted by ethanol in the tracer solution. Ethanol reduces the body temperature of mice [24], which might be compensated by glucose metabolism within the brown fat tissue. Additionally, ^18^F-fallypride striatum uptake was increased in animals receiving ethanol compared with that in control animals (16%, not significant). This trend supports the hypothesis that the rate of metabolic transformation of ^18^F-fallypride might be slowed down under acute ethanol conditions. It was assumed that ^18^F-fallypride is metabolized via an oxidative process in the liver. Acute ethanol administration is known to induce hypoxia in the liver [25] and is therefore believed to impact the enzyme capacity of oxygenating enzymes. Ethanol was therefore expected to slow down the degradation process of ^18^F-fallypride, a low extraction compound, resulting in altered uptake characteristics in its target organs (mainly the striatum). Varying contents of ethanol in the ^18^F-fallypride solution might therefore lead to increased inter-animal variability of striatum uptake, which may be considered for an optimal tracer formulation in future experiments.

It was previously reported that overnight fasting was beneficial for small animal ^18^F-FDG study outcome due to reduced competition of glucose with ^18^F-FDG for cellular uptake [11,16,26,27]. For this study, it was assumed that fasting of animals might conduce positively to the experimental outcome in two ways. Besides increasing the overall organ uptake of ^18^F-FDG, it was expected that the variability of blood glucose levels of individual animals would be reduced crucially and a relatively uniform low glucose concentration would be reached. This was assumed to reduce the inter-animal variability of ^18^F-FDG tissue uptake. However, a pilot experiment showed that fasting did not lead to reduced blood glucose levels or reduced variability of blood glucose concentration (data not shown). One reason for this might be the short fasting duration of only 4 h that was used to fulfill the regulations of the local authorities. These findings superseded a dedicated experiment to study the influence of blood glucose levels on ^18^F-FDG tissue uptake. Fasting of animals prior to ^18^F-FDG was therefore omitted in this study.

The homogenization study according to a protocol by Richter and co-workers [7] suggests that a low intra‐group variability does not necessarily correspond to low intergroup variability as generally assumed [5,6]. The significantly higher false positive rate under homogenized conditions reported by Richter and colleagues were not confirmed, most probably due to the smaller scale of this study (4 group comparisons per homogenized and heterogenized setup in this study vs. 18 group comparisons per setup in the study of Richter and co-workers). Nonetheless, the results revealed a clear trend towards affirmation of the findings by Richter et al. [7]. Regarding the low power of this study as well as the fact that the setup mixed empirical with data-mining data, it is important to interpret our results with care. Mainly, the results indicate that homogenization of experimental setup might not only be a problem in behavioral studies, but also in molecular imaging. This prompts more research in this regard as well as underlines the importance of careful interpretation and extrapolation of research results to the clinic. In the future, it might be worthwhile to investigate this topic by including different research centers to generate experimental heterogenization.

This study also shows that the variability of ^18^F-FDG tissue uptake is strongly dependent on the tissue of interest. Brain uptake was rather stable with a variability ranging from 6% to 16%, whereas the variability of ^18^F-FDG concentrations in blood and the Harderian gland was in a range of 20% to 40%. Considering this high variability for ^18^F-FDG organ uptake, it is advisable to perform sample size calculations prior to experiments to avoid exclusion of effects due to unsatisfactory study power. Furthermore, it shows the range of effect size that can still be detected with small animal PET without being lost in evitable variability.

## Conclusions

Small animal PET studies should be designed with care, taking into account that using animals as their own control does not necessarily increase reliability of results. Furthermore, experimental parameters which do not impair tracer distribution significantly (e.g., ambient temperature, housing conditions, gender, and age of animals) are suggested to be varied in a controlled manner to produce results with maximal external validity. In the future, it would be worthwhile to perform studies involving different PET centers pursuing small animal imaging to investigate closer the external validity of the results.

## Competing interest

The authors declare that they have no competing interests.

## Authors’ contributions

MIM-K participated in the study design, carried out all the experiments, did the statistical analysis, and drafted the manuscript. SMA revised the manuscript critically for important intellectual content. MFA helped to adjust the manuscript to fit the journal guidelines and revised the manuscript critically for important intellectual content. PAS revised the manuscript critically for important intellectual content. MH participated in the study design, biodistribution studies, as well as small animal PET scans and revised the manuscript critically for important intellectual content. All authors read and approved the final manuscript.
